# Microstructure and Mechanical Properties of Welded Joints of 1.4462 Duplex Steel Made by the K-TIG Method

**DOI:** 10.3390/ma14247868

**Published:** 2021-12-19

**Authors:** Przemysław Zmitrowicz, Michał Kawiak, Paweł Kochmański, Jolanta Baranowska

**Affiliations:** 1 Faculty of Mechanical Engineering and Mechatronics, West Pomeranian University of Technology in Szczecin, 70-310 Szczecin, Poland; michal.kawiak@zut.edu.pl (M.K.); pawel.kochmanski@zut.edu.pl (P.K.); jolanta.baranowska@zut.edu.pl (J.B.); 2 JW Steel Construction Sp. z o.o., Sp. k., 71-836 Szczecin, Poland

**Keywords:** keyhole welding, duplex stainless steel, microstructure, mechanical properties

## Abstract

K-TIG (Keyhole Tungsten Inert Gas) method is a new, emerging welding technology that offers a significant acceleration of the joining process, even for very thick plates. However, its potential for welding of certain materials is still unknown. Particularly challenging are duplex steels as this technology does not allow the use of a filler material, which is crucial for these steels and for weld joint microstructure adjustment. In order to demonstrate the suitability of this technology for single-pass welding of 1.4462 duplex steel detailed studies of the microstructure of the weld joints obtained for different linear energies were carried out and discussed with respect to their mechanical properties. According to the results obtained, the heat-affected zone (HAZ) shows a microstructure similar to the HAZ of duplex steel welded with the traditional TIG multi-pass methods. However, the weld, due to the lack of filler material, had a microstructure different to that typical for duplex steel welded joints and was also characterized by an increased content of ferrite. However, all joints, both in terms of microstructure and mechanical properties, met the requirements of the relevant standards. Moreover, the K-TIG process can be carried out in the linear energy range typical of duplex steel welding, although further optimization is needed.

## 1. Introduction

Duplex steels are characterized by a ferritic-austenitic microstructure, in this way combining the advantages of both chromium ferritic steel and chromium-nickel austenitic steel such as: high strength, good correlation of impact toughness with elongation, and excellent corrosion resistance in both gas and liquids environments [1,2,3,4]. They are used mainly in the chemical, petrochemical, paper, gas and oil mining and energy industries in aggressive environments. An important aspect of using duplex steel in the vast majority of applications is the possibility of welding [5,6]. However, reheating of the material to high temperatures during the welding process activates diffusion processes that influence the austenite-ferrite proportion in duplex steel weld joints, which could negatively affect their plasticity. An additional limitation of the weldability of duplex steel is its sensitivity to precipitation associated with secondary thermal cycles, leading to destabilization of the primary phases. These precipitates can take the form of secondary solutions, carbides, carbonitrides and other intermetallic phases. There are many precipitates that deteriorate the properties of this steel, the occurrence of which is conditioned by a sufficiently long stay of the steel within a specific temperature range. Particularly harmful is the intermetallic *σ* phase, which causes brittleness and lowers the corrosion resistance of the steel [7,8,9]. Duplex steels can be welded with all typical methods of arc welding (processes 111, 121, 135, 136, 141 according to ISO 4063 [10]; respectively also marked as MMA, SAW, MAG, FCAW, TIG) as well as laser and plasma [11]. The selection of the method should ensure that the required mechanical and corrosive properties of the joint are obtained with the highest possible efficiency of the process. Therefore, the selected technology needs to guarantee the achievement of a weld structure with an appropriate proportion of ferrite, without the precipitation of intermetallic phases. To this end, numerous technological requirements have to be met such as adding properly selected filler material to maintain a high level of austenite in the weld, the use of larger beveling angles than for austenitic steels and to increase the distance (gap) between the welded element (which, unfortunately, increases the number of layers in the weld joint), proper selection of linear welding energy, and thorough control of the interpass temperature to avoid changes in ferrite content and intermetallic phases precipitation. These requirements apply to all the above-mentioned welding methods used for duplex steels, and they become more problematic the thicker the welded plate is. They also complicate the process of qualifying the process itself and obtaining a welded joint with good mechanical properties and corrosion resistance. The solution to these problems may be the use of the K-TIG (Keyhole Tungsten Inert Gas) welding method for joining these steel grades.

K-TIG process is an innovative welding method; it is a variation of the TIG process that enables deep penetration of the arc, while ensuring high productivity and quality of the welding process. Due to the relatively low linear welding energy, this process has been used in the welding of such materials as: titanium and its alloys, zirconium, corrosion-resistant steels, and high-strength carbon steels [12,13,14]. The K-TIG process enables butt-welding of joints with full remelting, without beveling the edges, up to a thickness of 11 mm for corrosion-resistant steels, up to 16 mm for titanium welding and up to 9 mm for C-Mn carbon steels [15,16,17,18]. Such joints can be made as one-sided, single-pass welds without the use of any additional material for welding. The limitation, due to the complex and non-linear physics of the process, the K-TIG method can be used to connect both sheets as well as pipes, but only in the flat (horizontal) position.

Although this technology is very promising due to the efficiency of the welding process, it is still not widespread in industrial practice. First studies have shown its suitability for the welding of steel S32101 (lean duplex) [19,20,21]. Nevertheless, the implementation of this technology in industrial practice, especially for the welding of “difficult” steels such as duplex steels, requires a number of further studies. Particularly important is the study of the microstructure of the welded joint and its relation to mechanical properties, especially impact strength at low temperature.

The aim of this study was to investigate the microstructure of welded joints of duplex steel of 1.4462 grade with a thickness of 10 mm obtained by the K-TIG method at various linear welding energies and to discuss the effect on mechanical properties, including in particular impact toughness at −40 °C. The latter is significant from the viewpoint of the transition temperature to the brittle state, which is related to the permissible negative operating temperature of components made from these steels.

## 2. Materials and Methods

### 2.1. Material

A 10 mm thickness plate of 1.4462 grade according to EN 10088-2 [22] was used for the tests. The chemical composition of the base material (BM) is given in Table 1, and the mechanical parameters in Table 2.

### 2.2. Welding Procedure

The welding process was carried out using the K-TIG method on a HTIG-1000 device (Foreweld, Guangzhou, China) with a welding current adjustment range: 60–1000 A, voltage U0 = 79 V and 100% duty cycle for the maximum welding current. The power source was connected by control cables to a welding head with a tungsten electrode having a diameter of *φ* = 6.4 mm, and the whole system was cooled by a liquid in a closed circuit.

**Figure 1:**
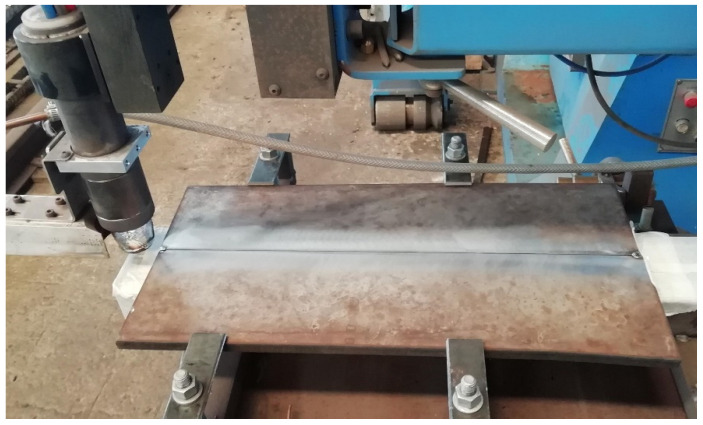
Preparation for welding.

Butt joints were made with full penetration—single-pass; one-side without a backing (BW ss nb), without beveling the joined elements (plates) and without spacing (gap) between them, and without the use of additional welding material (filler material). The shielding gas used was: argon—both on the side of the cap and the root of the weld. The preparation of elements for welding at the workplace is shown in Figure 1.

Five samples joints were made at the welding stand with variable parameters: welding current (A), arc voltage (V) and welding speeds (mm/s). The welding parameters were selected in such a way as to obtain the widest possible range of the linear welding energy, within the range permissible according the theoretical values specified for welding duplex steels 0.5–2.5 kJ/mm [4,5,23]. The second condition was to define the technological limits of the application of the K-TIG method for welding duplex steel with a thickness of 10 mm, without beveling and without the use of an additional material (filler material). The theoretical values of the linear welding energy were converted, based on Formula (1), into numerical values for individual welding parameters, assuming proper physics for the K-TIG process. In this way, five levels linear welding energy (kJ/mm) were obtained, the list of which is presented in Table 3.

The linear welding energy was determined on the basis of the Formula (1), as calculated without the coefficient of thermal efficiency of the welding method k. A detailed explanation for the application of the k-factor is provided in the Supplementary Materials (SM).

(1) $$Q=\frac{U\times I}{V_{\mathrm{sp}}}\times 1000,$$

where:

U—arc voltage (V),
I—welding current (A),
V_sp_—welding speed (mm/s).

The energy parameters of the welding process mentioned in Formula (1) can directly influence the linear welding energy. When it is required to use a narrow linear welding energy range, accurate measurement and constant supervision of the energy parameters becomes crucial in obtaining a welded joint with appropriate mechanical properties and corrosion resistance [24,25,26].

After completion of the welding process, the obtained welds were assessed for the presence of welding non-conformities in accordance with the requirements of EN-ISO 5817 [27]. Samples numbers 1 and 5 did not meet the assumed quality level “B” regarding the occurrence of welding imperfections.

In the sample number 1, a non-compliance defined as incomplete root penetration was observed along the entire length of the joint. The obtained penetration depth of the sample number 1, with the parameters according to Table 3, was: 8.91 mm. After macroscopic examinations, the presence of incompatibility identified as a gas pore in the cross-section of the welded joint with characteristic dimensions 4.53 × 3.33 mm in the plane of the cross-section, was revealed. The results of the examination of the macrostructure of sample number 1 are shown in Figure S1 in the Supplementary Materials.

The technological parameters adopted for the welding process of sample number 5 were too high for obtaining an acceptable joint (weld). The weld was not obtained along the entire length of the test plate. Due to the excessively high parameters, melting of the edges of the test panels in the area of the plate thickness occurs and the formation of a welding imperfection called burn-through was observed.

The samples number 2, 3 and 4 met the assumed quality level “B” in accordance with the requirements of EN ISO 5817 [27]. The test samples were cut from these plates for mechanical (destructive) and metallographic tests in specific parts of the welded joint according to the guidelines of EN ISO 15614-1 [28].

### 2.3. Mechanical Tests

The tensile test was performed for transverse samples in accordance with the requirements of ISO 4136 [29]. The test was carried out at ambient temperature (+20 °C), with a speed of 10 mm/min, using samples with a rectangular cross-section. In order to determine the percentage of elongation after fracture, the initial length of the measuring section was assumed to be 60 mm. The tensile test was performed on an INSTRON 5585H (Instron, Norwood, MA, USA), range: 0–250 kN.

The bending test was performed according to standard EN-ISO 5173 [30]. Two types of flat samples were used: TFBB (transverse face bend test specimen) and TRBB (transverse root bend test specimen) were used. The bending diameter was 40 mm and the distance between rollers was 65 mm. The acceptance criterion was a minimum bending angle of 180°. The bending test was performed on the INSTRON 5585H (Instron, Norwood, MA, USA), range: 0–250 kN.

The Charpy impact test was carried out on the welded joints in accordance with the requirements of ISO 148-1 [31], with the use of V-notch test specimens (LabTest CHK450J-I; Labortech, Opava, Czech Republic). The test specimens were cut along the rolling direction of the plate and perpendicular to the weld axis, with a reduced samples of 7.5 mm. The location of the notch of the samples was adopted in accordance with the designation according to ISO 9016 [32]. The tests were carried out at the temperature of −40 °C on the weld (WM) and for the heat affected zone (HAZ), and taken 2 mm below the upper surface of the base material (BM).

The Vickers method was used for the hardness tests with a load of HV10, in accordance with the requirements of ISO 9015-1 [33]. The hardness range was determined across the welded joint on two measuring lines (rows), one on the side of the cap and the other on the side of the weld root, at a depth of 2 mm from the surface down to the thickness of the plate. The hardness was measured in the WM, in the HAZ and in the BM. The test load was assumed: 10 Kgf and the load time was 13 s for each row. A value of 290 HV10 was adopted as the acceptance criterion. The tests were carried out on the Vickers Hardness Tester LV700AT (Leco, St. Joseph, MI, USA).

### 2.4. Microstructural Examination

Macroscopic tests were carried out in accordance with ISO 17639 [34], showing the individual zones of the welded joints made: the HAZ, the fusion line (FL) and the WM, in order to detect and identify possible welding defects and non-conformities. The test specimens were prepared by grinding and polishing, and the primary and secondary structure was visualized by etching with Adler’s reagent (25 mL-H_2_O, 3 g-(NH_4_)_2_CuCl_4_·2H_2_O, 50 mL-HCl, 15g-FeCl_3_). The observation was performed using a Nikon EPIPHOT 200 optical microscope (Nikon Corporation, Tokyo, Japan) and the NIS-Elements BR software was used for image analysis (Nikon Corporation, Tokyo, Japan).

Microscopic examination of the welded joints was carried out according to the procedure described in the standard ISO 17639 [34]. The structure components were identified on the basis of morphological features such as appearance, shape, and color. The types of microstructure components in individual areas of the welded joint were determined by the visualization using color etching, with the Berah II reagent (48 g-NH_4_F·HF, 400 mL-HCl, 12 g-K_2_S_2_O_5_, 800 mL-H_2_O). The examination was performed using a Nikon EPIPHOT 200 optical microscope (Nikon Corporation, Tokyo, Japan). Quantitative analyzes of the surface proportions of individual microstructure components were performed using the NIS-Elements BR computer program (Nikon Corporation, Tokyo, Japan).

Analysis of the chemical composition of the components of the structure of welded joints in particular areas, such as the HAZ and the WM, as well as the examination of the fracture surfaces after the impact test, were performed using a scanning electron microscope. An FE-SEM SU-70 (Field Emission Scanning Electron Microscopy) microscope (Hitachi, Naka, Japan) was used equipped with an EDS (energy dispersive spectrometry) X-ray microanalysis spectrometer (NORAN™ System 7 from Thermo Fisher Scientific, Madison, WI, USA). The SEM-EDS analysis was performed at an accelerating voltage of 15 kV.

## 3. Results and Discussion

### 3.1. Microstructures of the Welded Joints

The test weld joints were made with the technological parameters, which are shown in Table 3. These parameters, mainly welding current and welding speed, directly affect the geometry of the welds obtained. The voltage of the welding arc also has an indirect influence, but this parameter is not regulated on the welding device. However, it can be influenced by the distance of the torch, more precisely the tungsten electrode, from the surface of the welded material. During welding test joints, the distance between the torch nozzle and the welded material was maintained at 2.5 mm through manual adjustment.

**Figure 2:**
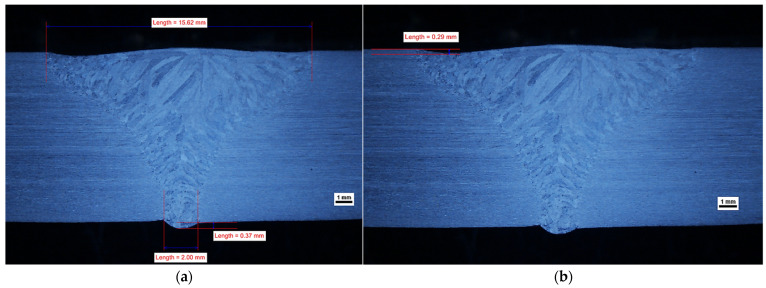
Effect of welding current on the geometry profile of weld (sample no 2): (**a**) geometry profile of weld with the welding current 533 A; (**b**) depth of undercut of weld.

**Figure 3:**
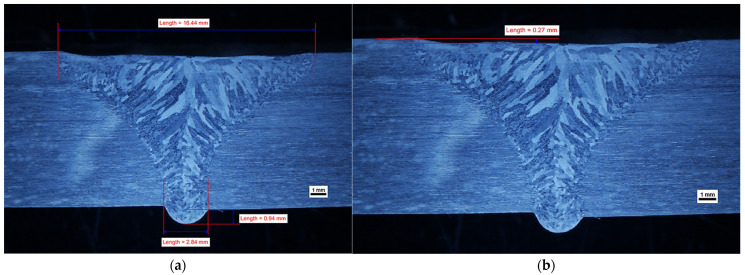
Effect of welding current on the geometry profile of weld (sample no 3): (**a**) geometry profile of weld with the welding current 583 A; (**b**) depth of undercut of weld.

**Figure 4:**
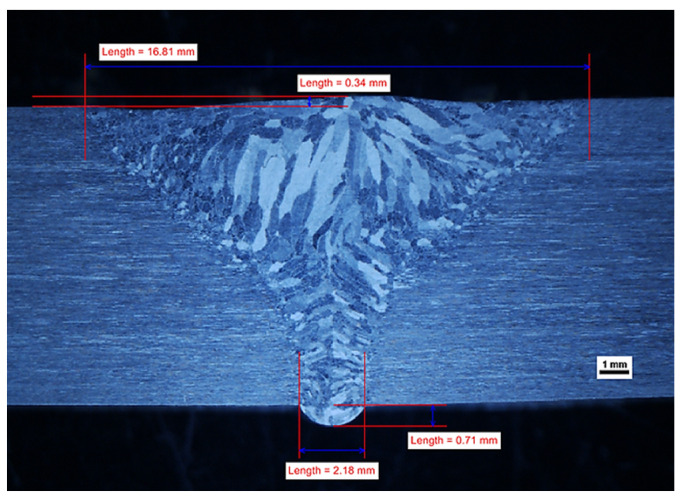
Effect of welding current 490 A on the geometry profile of weld (sample no 4).

The geometry of the obtained welds is shown in Figure 2, Figure 3 and Figure 4. The characteristic feature of all obtained welds is their “funnel” shape, which distinguishes the K-TIG from other arc welding methods.

In general, it can be assumed that if other welding parameters are constant, the width of the face (cap) and the fusion depth (penetration) increases with increasing welding current. At a level of 480 A, it is not possible to obtain a full penetration of the 10 mm thickness plate and there are gas pores in the weld (WM), as shown in Figure S1 in the Supplementary Materials. A similar phenomenon has been observed for lean duplex steel [19], for a welding current of 470 A and a welding energy of 2.215 kJ/mm. However, as the research carried out for lean duplex steel [19] has shown, it is not only the welding current that has an influence on the occurrence of this phenomenon. The formation of the “keyhole” also depends on the welding speed and a lack of penetration was also observed for a welding current of 530 A, when the welding speed was increased, thus decreasing the linear energy to 1.9 kJ/mm in the case of 10.8 mm plates [19]. If the thinner plates were welded, the higher welding speeds were acceptable (and the lower welding energies) [21]. In our research, when the welding current increased to 490 A, a complete penetration in the joint was reached with a correctly formed joint root, as shown in Figure 2, even though the linear energy was 2.18 kJ/mm; a value comparable to that for which there was a lack of fusion observed for lean duplex steel [19]. In our experiments, the welding speed was maintained below the harmful values of 4.5 mm/s [19], and no negative influence of this parameter was observed.

This experiment showed that the penetration depth of the welding arc was enhanced with increasing welding current, provided the welding speed was in a safety regime. At a low level of welding current, it was not possible to obtain a complete penetration of the 10 mm thick plate and there were gas pores in the WM. When the intensity of the welding current increased in order to create the “keyhole” effect, no gas pores were observed in the weld and the root obtained the correct shape. However, if the welding current intensity continued to increase to a level of 533–583 A, this upset the equilibrium between the static pressure of the molten metal in the weld pool and its surface tension [35]. This led to a collapse of the weld pool, causing the weld face profile to be too high in the axis of the weld, resulting in undercutting of one side. It should be noted that as the welding current increased, the “collapsing” of the weld pool caused a larger dimension of non-compliance in the form of undercutting, which is shown in Figure 2 and Figure 3. It is clear that a properly selected welding current intensity is the key to obtaining a correct profile of the weld on both the face and root sides.

On the obtained macrostructures, shown in Figure 2, Figure 3 and Figure 4, the characteristic areas of the welded joint are shown: the HAZ and the WM. The weld profile geometry was valid for all three joints. There is a smooth transition of the weld face to the BM and the root is properly formed. The size of non-conformities for samples number 2 and number 3, in the form of undercut, is acceptable for the adopted quality level “B” according to the standard EN-ISO 5817 [27]. There were no internal defects, non-conformities or irregularities in the structure of the HAZ and the BM. It is worth emphasizing that despite the lack of beveling of the plate before welding (preparation for I) and the solidification direction of the weld perpendicular to the direction of heat dissipation, the correct joint shape coefficient is ensured. This leads to a situation where the impurities are led to the top of the weld and not closed within a part of the molten metal in the center of the weld. In each of the welded joints, the size and configuration of the HAZ is similar. The average width of the HAZ in samples number 2, 3 and 4 was approximately 200 μm, narrower than the 300 μm typical for standard methods of arc welding of duplex steel [5].

Using an optical microscope, it was clearly visible with sample numbers 2, 3 and 4 that the microstructure consisted of two phases: austenite (light phase) and ferrite (dark phase). The structure can be considered as austenite fields (islands) against a background of ferritic matrix.

**Figure 5:**
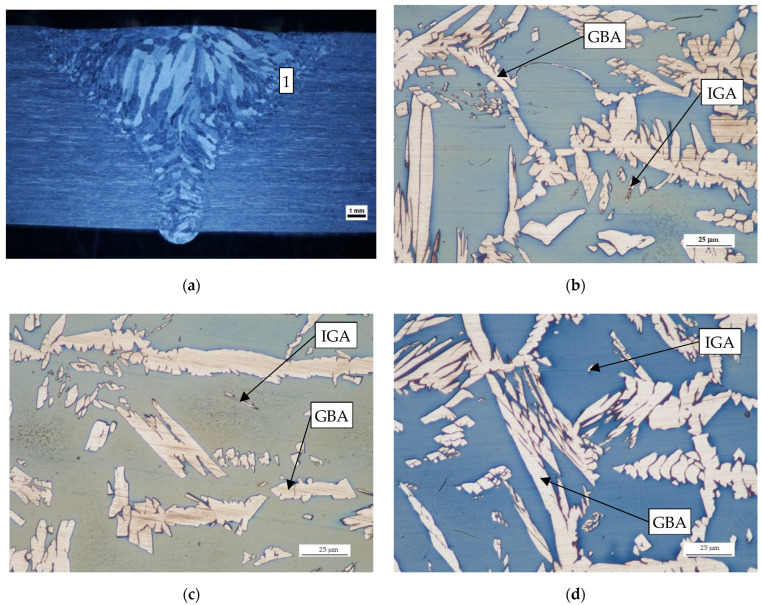
Microstructure of the HAZ: (**a**) transverse cross section of weld; (**b**) sample no 2; (**c**) sample no 3; (**d**) sample no 4.

**Figure 6:**
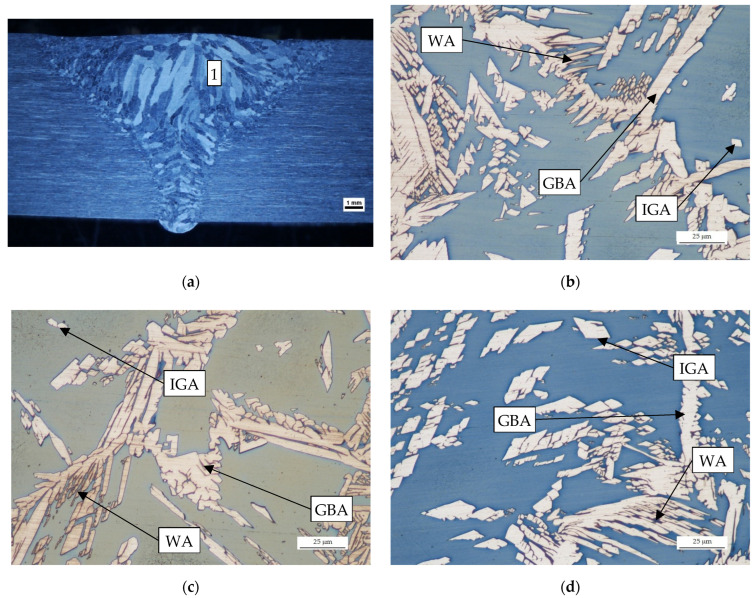
Microstructure of the WM: (**a**) transverse cross section of weld; (**b**) sample no 2; (**c**) sample no 3; (**d**) sample no 4.

The HAZ of the tested welded joints are shown in Figure 5, and the microstructure of the WM in Figure 6. In the K-TIG welding process, the arc energy is more concentrated than in other welding methods, therefore the HAZ zone is very much narrower. Under the influence of the thermal welding cycle, part of the austenite in the HAZ was deformed and it is clearly visible that this led to the destabilization of the primary phases. The ferrite grains have a coarse-grained structure, and some austenite has been dissolved in them. During the cooling process, austenite grains begin to precipitate at the ferrite grain boundaries so that the primary areas of austenite formation are along the ferrite grains, leading to grain boundary austenite (GBA)—Figure 5. As the transformation progresses, the grain boundaries are completely covered with austenite. It can also be observed that austenite grains are formed inside the ferrite grains, leading to intragranular austenite (IGA).

As shown in Figure 6, there are many thick columnar structures in the WM. The microstructure of this zone consists of grain boundary austenite (GBA) and intragranular austenite (IGA) as well as an austenite having a Widmanstätten (WA) structure in a ferrite matrix.

No intermetallic phases were detected in either the HAZ (Figure 5) or the WM (Figure 6).

**Figure 7:**
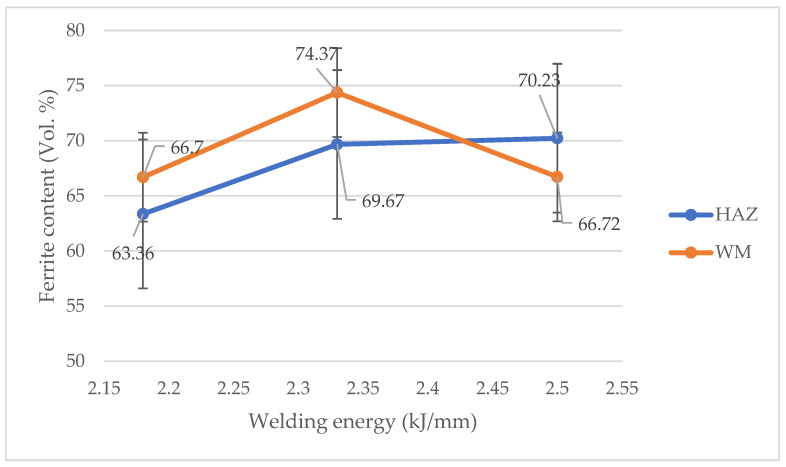
Ferrite volume fraction in the HAZ and WM of the welded joints.

Figure 7 shows the relative content of ferrite for individual welded joints in the HAZ and in the WM. It can be seen that for all samples the relative ferrite content is in the upper acceptable limit of 60–75%. As the linear welding energy increased, the ferrite content in the HAZ increased. In contrast, for the WM, the highest proportion of ferrite was measured for sample number 3. The proportion of ferrite in the microstructure can be controlled by monitoring the chemical composition and/or thermal cycles. The conducted experiment shows that the cooling rate at linear welding energies, even at a level of 2.50 kJ/mm, is so low that there is not enough time for a full diffusion-controlled phase transformation of ferrite to austenite. The high content of ferrite in the WM also results from the fact that the welding technology used did not require any additional material (filler material). The latter usually has an increased nickel content in relation to the parent material (BM) in order to obtain a greater content of austenite during weld cooling [5,36].

In the studies of the welding of lean duplex steel [20], it was observed that an increase in the linear energy of welding causes an increase in the proportion of austenite in the HAZ. This was explained by a slower cooling rate and thus an increase in the time necessary for diffusion-controlled phase transformation. In our study, the opposite relationship was observed (Figure 7). However, it should be noted that in [20], the increase in linear energy was directly proportional to the welding current, because the tests were carried out at a constant welding speed. In our case, similar values of linear energy were obtained by adjusting both parameters influencing the formation of the “keyhole”, i.e., welding current and welding speed. Assuming that the actual amount of heat introduced to the joint during welding determines the rate of phase transformations taking place both in the heat-affected zone and in the weld, the difference between our results and those presented in [20] suggests that the Formula (1) does not correctly reflect the amount of heat introduced during the K-TIG welding process. However, this hypothesis requires a number of further studies in order to be confirmed.

**Figure 8:**
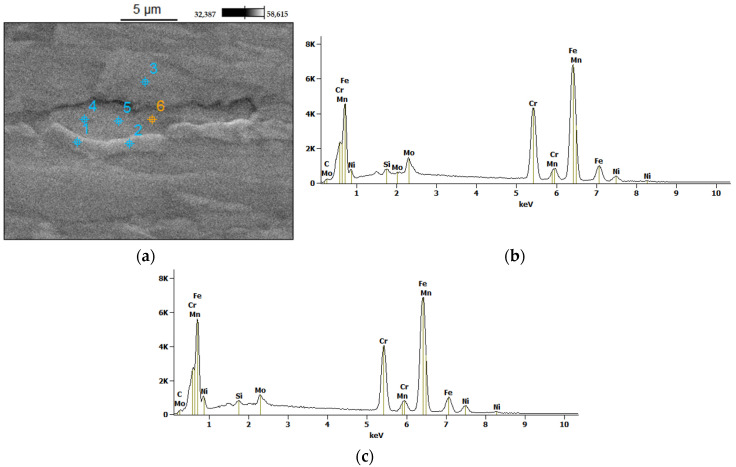
Microanalysis of the base material (EDS, SEM): (**a**) example of the measuring points; examples of energy spectra (**b**) for ferrite (point no 6); and (**c**) for austenite (point no 1).

SEM analyses were carried out on samples 2, 3 and 4 in the HAZ and the WM. For comparison, the BM for sample number 4 was additionally analyzed. Two components present in the duplex steel were tested: austenite (convex area) and ferrite (flat area), as shown, for example, in Figure 8.

The measured values of the Cr, Mo and Ni content in austenite and ferrite grains for all tested samples are presented in Table S1 included in Supplementary Materials. Measurements of the element content in austenite and ferrite in the BM confirmed the distribution of elements typical for duplex steel. A higher chromium content (23.5 wt%) occurs in ferrite, compared to in austenite (21.0 wt%). This is because chromium is a ferrite-forming element and diffuses into the ferrite. The nickel content is higher in the austenite than in the ferrite: 6.9 wt% and 4.8 wt%, respectively, because nickel is an austenite-forming component and diffuses into austenite under equilibrium conditions. Molybdenum, as a ferrite-forming element, is present in a greater amount in ferrite than in austenite, where its average content is respectively: 3.5 wt% and 2.3 wt%.

**Figure 9:**
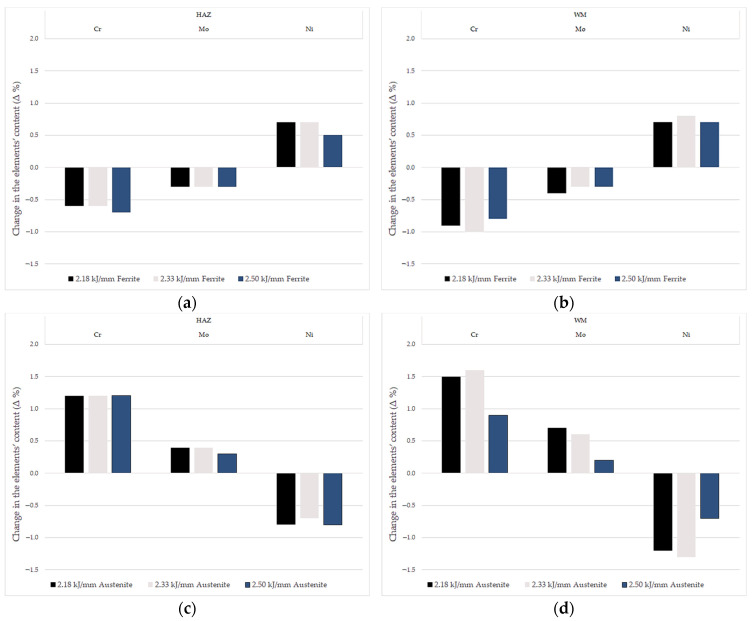
Chemical composition of the different phases, EDS: (**a**) ferrite in HAZ; (**b**) ferrite in WM; (**c**) austenite in HAZ; (**d**) austenite in WM.

However, significant differences were observed for both the HAZ and the WM in the chemical composition of the austenite and ferrite in comparison to the BM, as shown in Figure 9. In the HAZ zone, the content of chromium in the ferrite and austenite differed only slightly, regardless of the magnitude of the linear welding energy. It should be noted that the content of chromium in ferrite was lower, and in austenite higher, than that measured in the BM (Figure 9). A similar relationship is observed for molybdenum. In contrast, the nickel content in austenite is lower and in ferrite higher than in the parent material.

In the WM, as in the HAZ, the level of chromium content in both phases is balanced, and for all elements their amounts are too high or too low compared with the parent material. As can be seen from the results obtained, both in the case of the HAZ and WM, the amount of basic alloying elements in ferrite and austenite differs from those measured in the base material. This is most likely due to the limited possibility of diffusion of these elements during cooling. It is noteworthy that this effect is independent of the linear welding energy parameter within the range of 2.18–2.50 kJ/mm.

### 3.2. Mechanical Properties of Welded Joints

**Figure 10:**
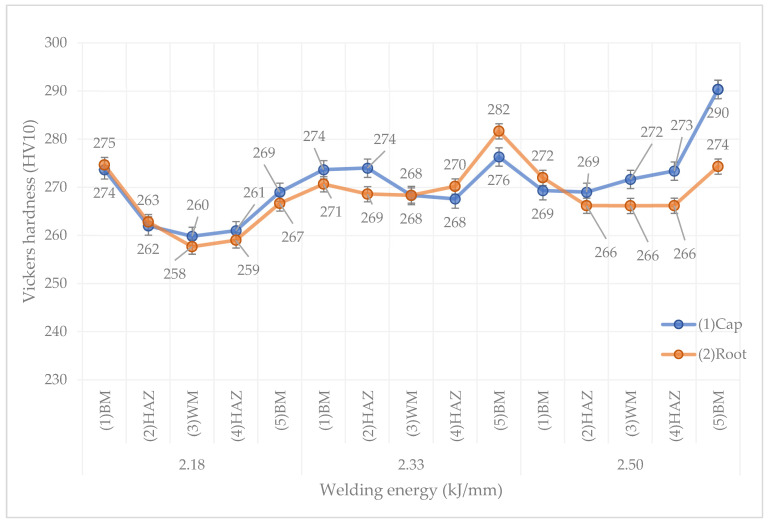
Hardness distribution in the welded joints.

Figure 10 shows the average values of HV10 hardness in the individual characteristic zones of the welded joint. The obtained hardness for all test joints in each of their zones meets the requirement of 290 HV10.

The hardness distribution for individual test joints is almost symmetrical. It can be clearly observed that the hardness of the HAZ is similar to the hardness of the WM, and this may be due to the fact that the relative volume of ferrite in these two zones is at a similar level. There is also a tendency for the hardness to increase in the joint with increasing linear welding energy, probably due to a noticeable increase in the ferrite content in the individual zones of the samples number 3 and 4 in relation to the samples number 2 (Figure 7). Moreover, no significant differences were observed between the hardness measured from the cap and from the root sides, despite such a thick plate.

**Figure 11:**
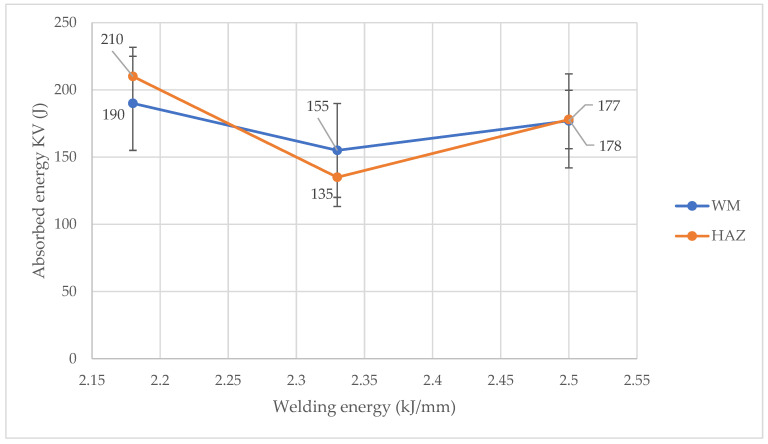
Impact properties of the welded joints.

Impact tests were performed using the Charpy method for reduced samples (7.5 mm) at the temperature of −40 °C. Figure 11 summarizes the obtained absorbed energy in the HAZ and in the WM at different values of the linear welding energy.

**Figure 12:**
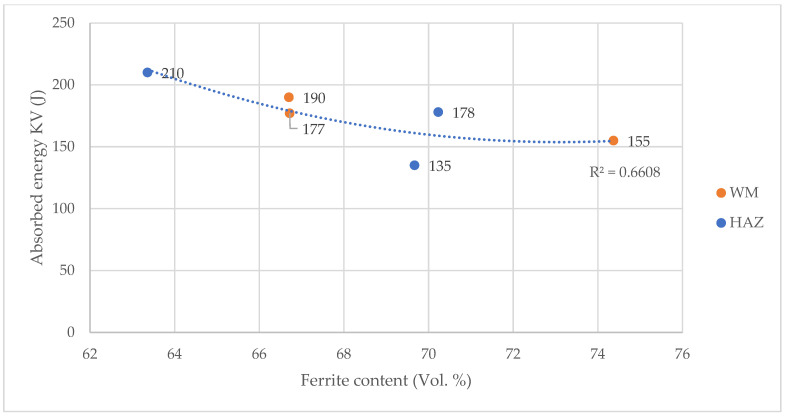
Dependence of absorbed energy on the ferrite volume.

The results show (Figure 11) that the absorbed energy is the highest in the case of the samples number 2, with a linear welding energy value of 2.18 kJ/mm. The lowest absorbed energy was obtained for the samples number 3, for which the linear welding energy was 2.33 kJ/mm. In general, the absorbed energy obtained for all joints did not differ significantly from each other and all meet the assumed acceptance criteria of 33 KV_2_. Nevertheless, the observed minimum absorbed energy for sample number 3 corresponds to the increased amount of ferrite observed in this sample (Figure 7), so there is a clear relationship between the microstructure of the weld joints and their impact properties as shown in Figure 12. However, as it was already stated, a thorough explanation of the observed phenomena needs further studies.

**Figure 13:**
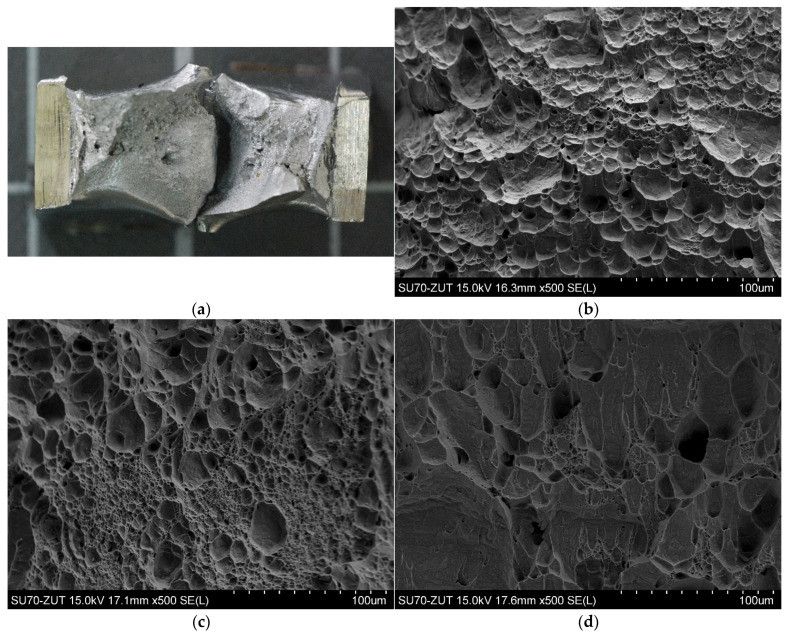
SEM morphologies of fracture surface in the HAZ (**a**) fracture of the impact test piece (**b**) sample no 2; (**c**) sample no 3; (**d**) sample no 4.

**Figure 14:**
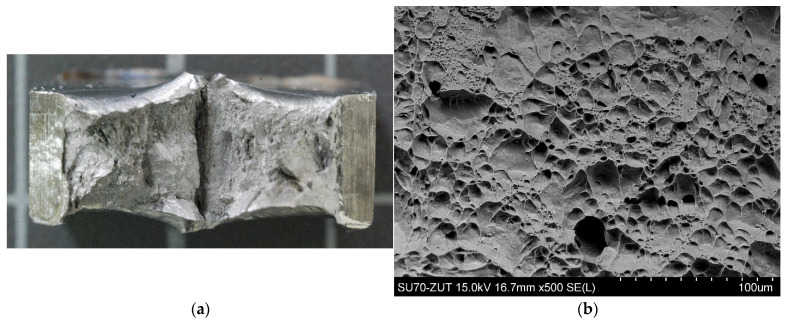

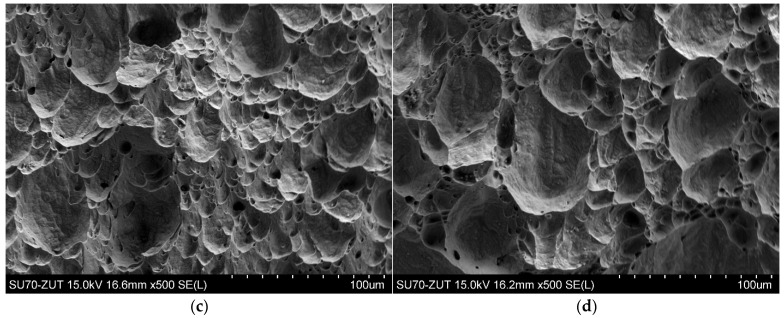
SEM morphologies of fracture surface in the WM (**a**) fracture of the impact test piece (**b**) sample no 2; (**c**) sample no 3; (**d**) sample no 4.

The nature of the fractures obtained after the impact test for samples number 2, 3 and 4 in the HAZ is shown in Figure 13, and in the WM in Figure 14. From the observation of the fracture surface, it can be concluded that independently of the amount of linear welding energy within the limits studied, the cracking is transcrystalline, spreading through the grains and ductile areas and occurring by shearing of the crystal in the sliding planes under the influence of tangential stress. The fractures in the observed samples are matte and fibrous.

The detailed results of the tensile test are shown in the Table S2 presented in Supplementary Materials. All the tested joints met the requirements of the base material (BM). These requirements for the yield strength (*Rp0.2*): at least 480 MPa, the tensile strength (*Rm*): in the range of 680–840 MPa and the elongation (*A*): minimum 25%.

**Figure 15:**
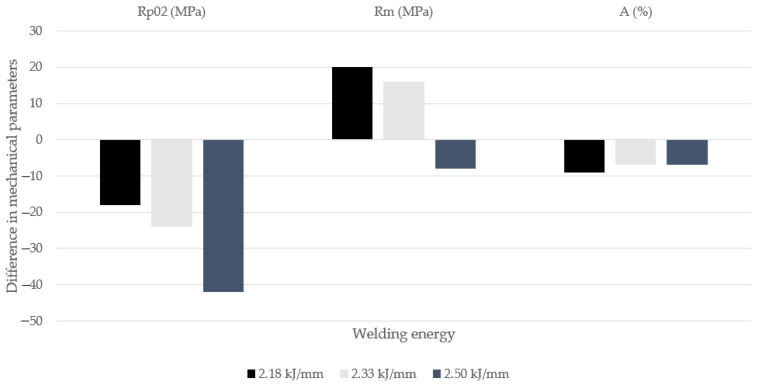
Changes in the strength parameters of the obtained joints in comparison to the parent material.

Figure 15 shows the changes in the strength parameters of the tested joints in relation to the parent material. For samples 2 and 3 welded with the linear energy, respectively 2.18 and 2.33 J/mm, a clear increase in the strength parameter (*Rm*) and deterioration of the plasticity parameters (*Rp0.2* and *A*) were observed. This maybe a results of the observation that during the cooling process ferrite was obtained predominantly as a component of the structure, which has greater hardness and lower plasticity compared to austenite.

On the other hand, the tensile strength decreased for sample 4 in relation to the value obtained for the base material. It should be emphasized that for this sample in the WM the ferrite content was slightly lower, and the content of Cr, Mo and Ni was the closest to the content measured for the BM. This would suggest that for this sample the cooling process was slow enough for the diffusion processes and phase changes to take place to a noticeable degree.

**Figure 16:**
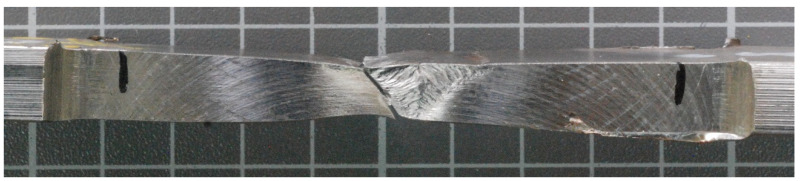
Fracture behavior—sample no 2.

**Figure 17:**
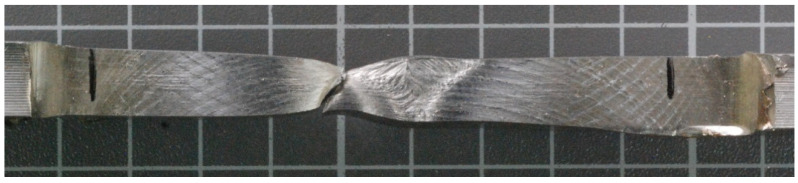
Fracture behavior—sample no 3.

Figure 16 and Figure 17 show, the fracture points after the tensile tests for sample numbers 2 and 3, respectively. For both samples, the break occurred in the location of the undercut, observed in the macrographic examination (Figure 2 and Figure 3). Being a typical geometric notch, a natural stress accumulation occurred at this location. The fracture angle is approximately 45°, which confirms the plastic nature of the fracture at the site of tangential stresses.

**Figure 18:**
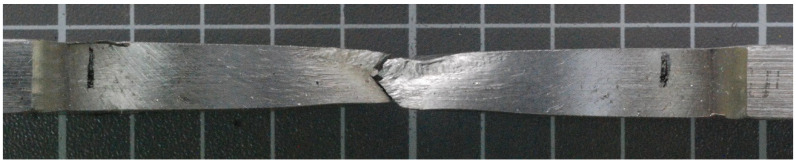
Fracture behavior—sample no 4.

The fracture point of the sample number 4 is shown in Figure 18 and shows that a crack occurred in the weld. In the macrographic examination, no undercut was found in this joint (Figure 4). The fracture was ductile, as evidenced by its matte and fibrous surface. It should be noted that the obtained tensile strength in the WM was lower than the tensile strength of the BM.

The ductility of samples number 2, 3 and 4 was confirmed by a bend test, as shown in Figures S2–S7 in the Supplementary Materials. The bending angle of 180° was obtained for all tested samples. Both for the tensile bending test of the weld face and for the tensile bending test of the weld root, no cracks were observed on the tensile surfaces, proving good ductility of the welded joints. There were no differences showing that within the tested range of 2.18–2.50 kJ/mm, the linear welding energy had a significant impact on the result of the bending test.

## 4. Conclusions

Weld joints with full penetration were successfully obtained on a 10 mm thick 1.4462 duplex plates using the single-pass K-TIG method, within a welding energy range of 2.18–2.50 kJ/mm. The microstructure of the weld was significantly different to that obtained during typical welding methods used for these steel grades due to the lack of additional materials (filler material), which are not used in the K-TIG method. However, although the welds were characterized by a relatively high ferrite content (67–75%), their mechanical parameters met the requirements for the base material (BM). In particular, it has to be stressed that also the value of the absorbed energy obtained during the impact test at a temperature of −40 °C, in all areas of the welded joint, also met these requirements as well.

Limited differences in the distribution of the main alloying elements between austenite and ferrite grains were observed within the range of the linear welding energy studied. This indicates that the cooling rate after welding was too high for both the effective diffusion of the alloying elements and the ferrite-austenite phase transformation.

It was observed that the welding current and welding speed significantly affect the geometry of the weld. This applies in particular to obtaining the appropriate shape of the weld cap without undercut and the correct shape of the root, i.e., obtaining a full penetration of the plate.

Differences were observed in the amounts of ferrite measured as a function of the linear welding energy in the tested joints compared to the literature data for lean duplex steel. This could not be explained on the basis of the current limited knowledge of the physics underlying the K-TIG welding method. Thus, there is a need for further extensive research on the influence of welding parameters on the microstructure of joints, in order to optimize these joints for practical implementation of this very promising method.

## Figures and Tables

**Table 1:** Chemical composition of welded steel [wt%].

C	Si	Mn	P	S	Cr	Ni	Mo	N
0.018	0.35	1.32	0.025	0.001	22.28	5.75	3.17	0.17

**Table 2:** Mechanical properties.

Yield Strength Rp0.2 [MPa]	Tensile Strength Rm [MPa]	Elongation A5 [%]	Impact Test KV (−40 °C) [J]	Ferrite Volume [%]
606	786	36	165	47

**Table 3:** Welding parameters and linear welding energy.

Test Piece No:	Welding Current I [A]	Arc Voltage U [V]	Welding Speed V_sp_ [mm/s]	Welding Energy Q [kJ/mm]
1	480	15.7	3.93	1.92
2	533	16.0	3.92	2.18
3	583	17.1	4.29	2.33
4	490	16.7	3.28	2.50
5	595	17.5	3.52	2.96

## Data Availability

Not applicable.

## Supplementary Materials

The following are available online at https://www.mdpi.com/article/10.3390/ma14247868/s1, Figure S1: Macroscopic picture of welded joint cross section of sample no 1, with no acceptable incomplete root penetration, Figure S2: Transverse face bend test specimen—sample number 2; (a) sample after bending; (b) surface of the tension side—cap of weld, Figure S3: Transverse root bend test specimen—sample number 2; (a) sample after bending; (b) surface of the tension side—root of weld, Figure S4: Transverse face bend test specimen—sample number 3; (a) sample after bending; (b) surface of the tension side—cap of weld, Figure S5: Transverse root bend test specimen—sample number 3; (a) sample after bending; (b) surface of the tension side—root of weld, Figure S6: Transverse face bend test specimen—sample number 4; (a) sample after bending; (b) surface of the tension side—cap of weld, Figure S7: Transverse root bend test specimen—sample number 4; (a) sample after bending; (b) surface of the tension side—root of weld, Table S1: Chemical composition of ferrite and austenite grains in different zones of welded joints, Table S2: Mechanical parameters determined from the tensile tests.
