# Supplementary material for: Microstructure and Mechanical Properties of Welded Joints of 1.4462 Duplex Steel Made by the K-TIG Method
Source: Materials (Basel). 2021 Dec 19;14(24):7868. doi: 10.3390/ma14247868 (PMC8708518; doi:10.3390/ma14247868)
Supplement: Supplementary file 1 [file materials-14-07868-s001.zip › materials-1507027-supplementary.pdf]

materials

MDPI

Supplementary Materials

Microstructure and Mechanical Properties of Welded Joints of 1.4462 Duplex Steel Made by the K-TIG Method

Przemysław Zmitrowicz [1,2,*], Michał Kawiak [1], Paweł Kochmański [1] and Jolanta Baranowska [1]

1 Faculty of Mechanical Engineering and Mechatronics, West Pomeranian University of Technology in Szczecin, 70-310 Szczecin, Poland; michal.kawiak@zut.edu.pl (M.K.); pawel.kochmanski@zut.edu.pl (P.K.); jolanta.baranowska@zut.edu.pl (J.B.)

2 JW Steel Construction Sp. z o.o., Sp. k., 71-836 Szczecin, Poland

* Correspondence: zp22185@zut.edu.pl

Citation: Zmitrowicz, P.; Kawiak, M.; Kochmański, P.; Baranowska, J. Microstructure and Mechanical Properties of Welded Joints of 1.4462 Duplex Steel Made by the K-TIG Method. *Materials* **2021**, *14*, 7868. https://doi.org/10.3390/ma14247868

Academic Editor: Bolv Xiao

Received: 28 November 2021

Accepted: 16 December 2021

Published: 19 December 2021

Publisher's Note: MDPI stays neutral with regard to jurisdictional claims in published maps and institutional affiliations.

Copyright: © 2021 by the authors. Licensee MDPI, Basel, Switzerland. This article is an open access article distributed under the terms and conditions of the Creative Commons Attribution (CC BY) license (https://creativecommons.org/licenses/by/4.0/).

Explanations Concerning K-Factor

In the paper the linear welding energy was determined on the basis of the Formula (S1), as calculated without the coefficient of thermal efficiency of the welding method k. This is due to the fact that in the standard EN 1011-1 [1], this coefficient for the K-TIG method was not specified, which is directly related to the physics of this process. It should also be mentioned that various publications have reported using the coefficient k = 0.9, assuming the efficiency of the K-TIG method at the theoretical level of welding with the laser and electron beam method, or k = 0.6 [2–4]. In other publications, the k coefficient was not specified. The lack of an unequivocal determination of the coefficient k of the thermal efficiency of the welding method make it difficult to unambiguously reference and compare the results of experiments in individual publications on the weldability of duplex steels. Since the parameter of linear welding energy is essential from the viewpoint of the mechanical properties and corrosion resistance of the joint, it was concluded that the inadequate specification of the k-factor would enable an unambiguous reference and comparison of the test results with the experimental values of the K-TIG welding process for duplex steel in this paper.

where:

U – arc voltage (V),

I – welding current (A),

V_{sp} – welding speed (*mm*/*s*).

$$Q = \frac{U \times I}{V_{sp}} \times 1000, \quad \text{(S1)}$$

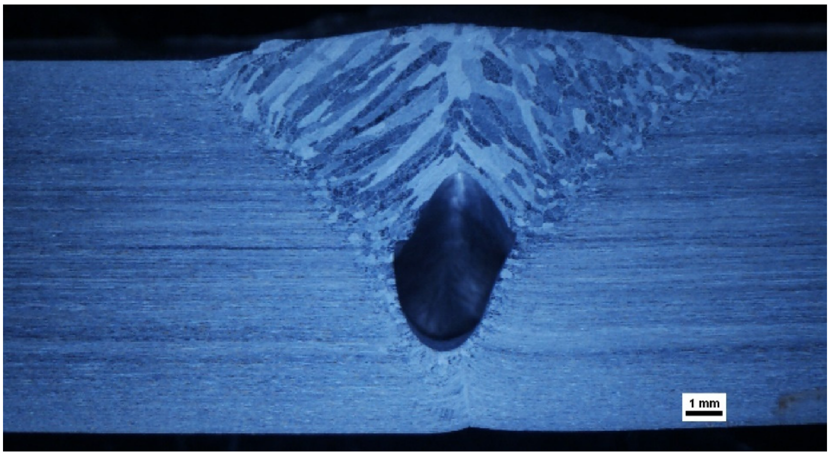

Figure S1. Macroscopic picture of welded joint cross section of sample no 1, with no acceptable incomplete root penetration.

Table S1. Chemical composition of ferrite and austenite grains in different zones of welded joints.

		Welding Energy					
		2.18 kJ/mm		2.33 kJ/mm		2.50 kJ/mm	
		Ferrite	Austenite	Ferrite	Austenite	Ferrite	Austenite
BM	Cr	23.5 ± 0.26	21.0 ± 0.22	23.5 ± 0.26	21.0 ± 0.22	23.5 ± 0.26	21.0 ± 0.22
	Mo	3.5 ± 0.09	2.3 ± 0.04	3.5 ± 0.09	2.3 ± 0.04	3.5 ± 0.09	2.3 ± 0.04
	Ni	4.8 ± 0.19	6.9 ± 0.33	4.8 ± 0.19	6.9 ± 0.33	4.8 ± 0.19	6.9 ± 0.33
HAZ	Cr	22.9 ± 0.25	22.2 ± 0.27	22.9 ± 0.30	22.2 ± 0.45	22.8 ± 0.28	22.2 ± 0.74
	Mo	3.2 ± 0.19	2.7 ± 0.19	3.2 ± 0.17	2.7 ± 0.06	3.2 ± 0.16	2.6 ± 0.23
	Ni	5.5 ± 0.19	6.1 ± 0.21	5.5 ± 0.35	6.2 ± 0.35	5.3 ± 0.37	6.1 ± 0.51
WM	Cr	22.6 ± 0.20	22.5 ± 0.15	22.5 ± 0.19	22.6 ± 0.22	22.7 ± 0.40	21.9 ± 0.32
	Mo	3.1 ± 0.00	3.0 ± 0.15	3.2 ± 0.12	2.9 ± 0.22	3.2 ± 0.09	2.5 ± 0.25
	Ni	5.5 ± 0.20	5.7 ± 0.15	5.6 ± 0.28	5.6 ± 0.34	5.5 ± 0.42	6.2 ± 0.43

Table S2. Mechanical parameters determined from the tensile tests.

Welding Energy Q (kJ/mm)	Yield Strength Rp0.2 (MPa)	Tensile Strength Rm (MPa)	Elongation A5 (%)	Location of Fracture
2.18	588 ± 11.7	806 ± 5.3	27 ± 1.2	HAZ
2.33	582 ± 25.5	802 ± 1.1	29 ± 1.0	HAZ
2.50	564 ± 18.4	778 ± 10.2	29 ± 1.0	WM

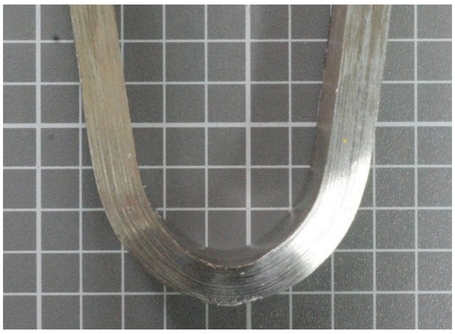

(**a**)

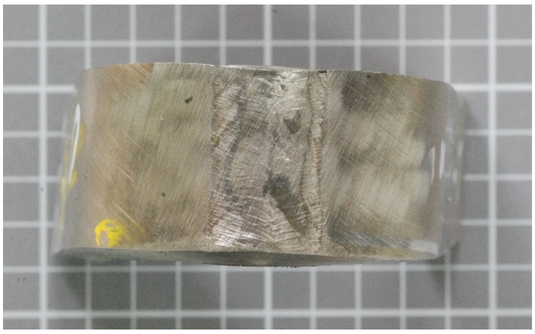

(**b**)

Figure S2. Transverse face bend test specimen – sample number 2; (**a**) sample after bending; (**b**) surface of the tension side – cap of weld.

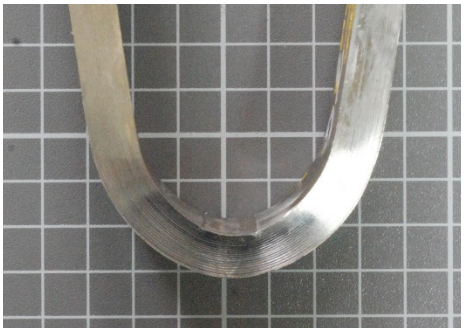

(**a**)

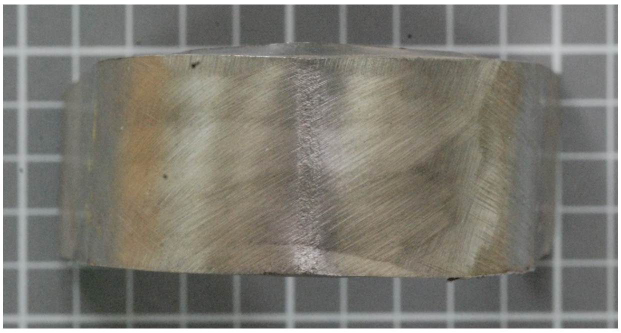

(**b**)

Figure S3. Transverse root bend test specimen – sample number 2; (**a**) sample after bending; (**b**) surface of the tension side – root of weld.

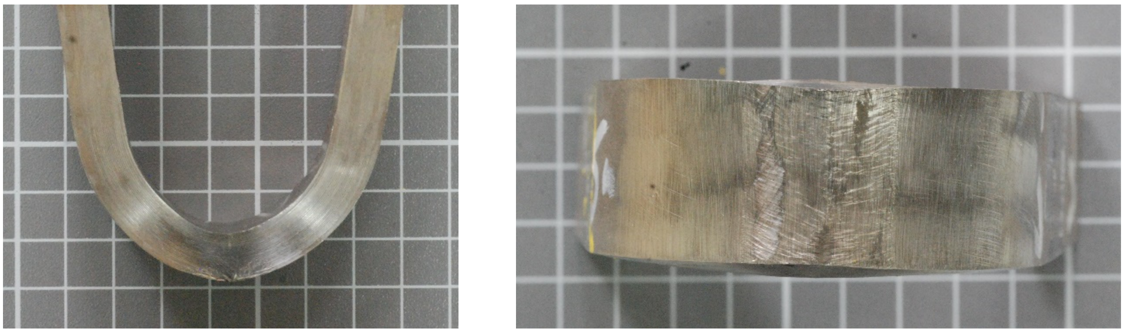

(**a**) (**b**)

Figure S4. Transverse face bend test specimen – sample number 3; (**a**) sample after bending; (**b**) surface of the tension side – cap of weld.

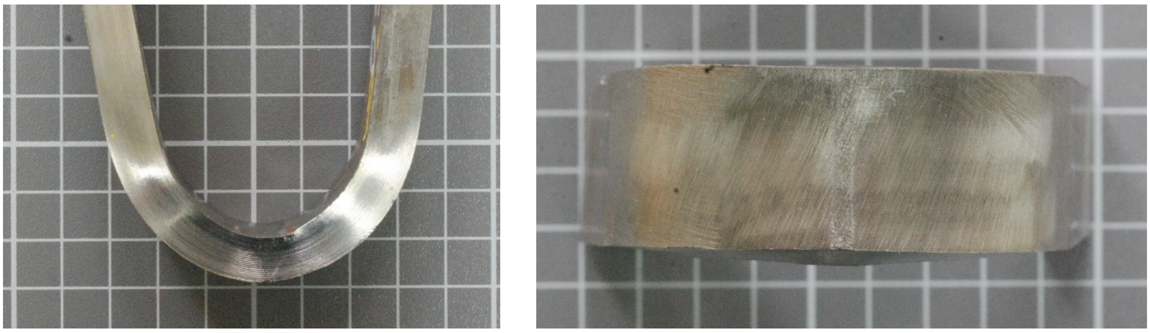

(**a**) (**b**)

Figure S5. Transverse root bend test specimen – sample number 3; (**a**) sample after bending; (**b**) surface of the tension side – root of weld.

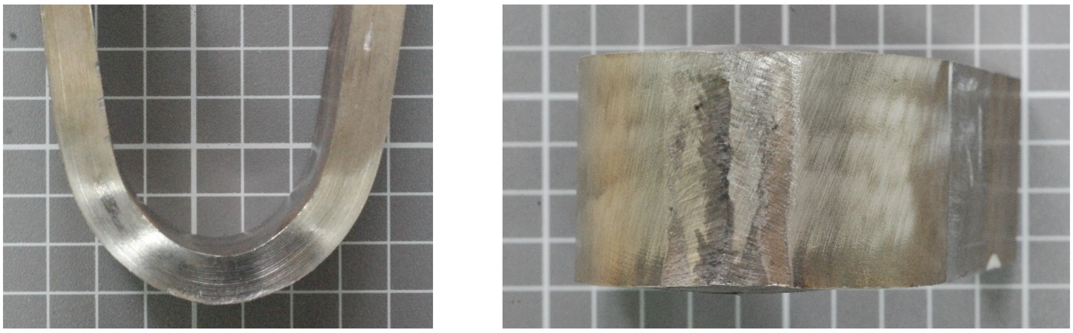

(**a**) (**b**)

Figure S6. Transverse face bend test specimen – sample number 4; (**a**) sample after bending; (**b**) surface of the tension side – cap of weld.

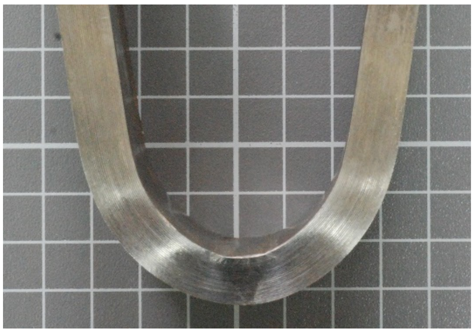

(**a**)

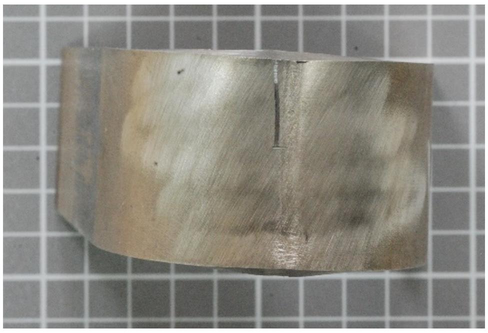

(**b**)

Figure S7. Transverse root bend test specimen – sample number 4; (**a**) sample after bending; (**b**) surface of the tension side – root of weld.

References

1. EN 1011-1: 2009 Welding-recommendations for welding of metallic materials-Part 1: General guidance for arc welding
2. Shi, Y.; Cui, S.; Zhu, T.; Gu, S.; Shen, X. Microstructure and intergranular corrosion behavior of HAZ in DP-TIG welded DSS joints. *Journal of Materials Processing Tech.* **2018**, *256*, 254–261
3. Liu, Z.M.; Chen, S.Y.; Liu, S.; Luo, Z.; Yuan, J.R. Keyhole dynamic thermal behavior in K-TIG welding process. *International Journal of Heat and Mass Transfer* **2018**, *123*, 54–66
4. Fei, Z.; Pan, Z.; Cuiuri, D.; Li, H.; Wu, B.; Ding, D.; Su, L. Effect of heat input on Weld formation and tensile properties in keyhole mode TIG welding process. *Metals* **2019**, *9*, 1327
